# Rs4911154 of circ-ITCH aggravated tumor malignancy of thyroid nodules via the circ-ITCH/miR-22-3p/CBL axis

**DOI:** 10.1038/s41598-021-97471-5

**Published:** 2021-09-16

**Authors:** Yiqing Guo, Hua Zheng, Jie Yin, Huaming Wang

**Affiliations:** 1 General Surgery, Gansu Second People’s Hospital, Lanzhou, 730000 Gansu China; 2 Ultrasonography Department, Lanzhou Maternal and Child Health Care Hospital, No. 74 Wuquan West Rd, Lanzhou, 730030 Gansu China; 3 Institute of Anatomy and Embryology, School of Basic Medical Sciences, Lanzhou University, Lanzhou, 730000 Gansu China; 4 Department of Pediatric Orthopedics, Gansu Provincial Hospital of TCM, Lanzhou, , 730050 Gansu China

**Keywords:** Cell biology, Molecular biology

## Abstract

Recent evidence revealed an inhibitory effect of circ-ITCH on the progression of papillary thyroid cancer via affecting the circ-ITCH/miR-22-3p/CBL axis. Rs4911154, an SNP located in circ-ITHC, was previously reported to be significantly associated with an increased risk of hepatocellular carcinoma. Ultrasound testing was used to evaluate the doubling time of thyroid nodules. 202 patients diagnosed with thyroid nodule disorders were divided into three groups according to their genotypes at rs4911154. We found that the A allele was correlated with a shortening doubling time of thyroid nodules. Moreover, the A allele contributed to reduced expression of circ-ITCH/CBL and increased expression of miR-22-3p. Besides, decreased tissue apoptosis was linked to the A allele. Luciferase assays indicated that miR-22-3p could effectively suppress the luciferase activities of CBL and circ-ITCH. Furthermore, manual up-regulation of miR-22-3p effectively suppressed the expression of CBL, while CBL siRNA apparently abolished circ-ITCH induced CBL upregulation, reduced proliferation and increased apoptosis of K1 and TPC-1 cells. A signaling pathway of circ-ITCH/miR-22-3p/CBL axis was established to explain the effect of SNP of circ-ITCH in thyroid tumor malignancy. Compared with the G allele, the A allele in rs4911154 contributed to the malignancy of thyroid nodules with decreased doubling time and down-regulated CBL expression.

## Introduction

Thyroid nodule (TN) is common while its incidence can be as high as 65% in the general population^1,2^. While TN is often diagnosed, the primary clinical risk of TN is actually the possibility of malignant transformation (the incidence of malignant transformation of TN can be as high as 6.5%^3^. Previous researches have presented that cytologically benign and asymptomatic TN commonly show almost no development when followed closely^4^. Thus, TN doubling time is an effective indicator used for the evaluation of TN malignancy.

Circular RNA (circRNA) belongs to a type of RNAs acting as possible diagnostic biomarkers by playing crucial roles in the progression as well as development of many cancers^5–9^. It was actually disclosed that circRNAs can play an important function in inducing malignant phenotypes to affect the apoptosis, invasion, vascularization, and metastasis of cancer cells^6,10,11^. It was indicated using bioinformatics tools that circRNA circ-ITCH can target miR-22-3p. With dual-luciferase assays, it was shown that circ-ITCH could indeed bind to miR-22-3p. qPCR assays presented that the overexpression of circ-ITCH reduced miR-22-3p expression in PTC cells. Additionally, the expression of circ-ITCH and miR-22-3p showed an inverse correlation, and miR-22-3p promoted cell expansion while hindering cancer cell apoptosis^12^. With bioinformatics tools, it was determined that Casitas B-lineage Lymphoma (CBL) gene acted as a miR-22-3p target, and both luciferase and qRT-PCR assays illustrated the interaction between CBL and miR-22-3p. Also, it was uncovered that circ-ITCH upregulated CBL expression through hindering miR-22-3p expression^13^.

The CBL protein can moderate the activity of tyrosine kinases through ubiquitylation. Upon tyrosine kinase ubiquitylation induced by CBL, nonreceptor and receptor tyrosine kinases undergo lysosomal degradation^14–16^.Therefore, CBL protein plays a vital role to control tyrosine kinase pathways and affect critical cellular processes like cell development as well as cell–cell communication^17^. In humans, all three types of CBL, i.e., c-CBL, b-CBL, as well as CBL-c, share a same tyrosine kinase binding (TKB) domain consisted of a phosphotyrosine binding SH2 domain as well as a RING domain responsible for moving ubiquitin from E2 proteins onto lysine residues.

In one research, it was shown that circ-ITCH hinders the activation of the Wnt/bcatenin signaling by upregulating the expression of CBL. Additionally, CBL downregulation significantly impaired the impact of overexpressed circ-ITCH on the proliferation, apoptosis and invasion of PTC cells, therefore indicating that CBL can inhibit PTC development^13^. In another research, it was discovered that rs10485505 as well as rs4911154 SNPs in circ-ITCH was substantially linked to elevated HCC risk^18^. These results point to potential genetic HCC determinants located in circRNAs. In one past research, tissue samples were collected from patients TT, TG, and GG genotypes of a key SNP, and it was found that TN doubling time was longer in the TT + TG group as compared with TN doubling time in the GG group. Also, let-7 g expression in TT, TG, as well as GG groups remained the same, while KRAS expression in the GG group was much higher than that in TT as well as TG groups. Rs4911154, an SNP located in circ-ITHC, was previously reported to be significantly associated with an increased risk of hepatocellular carcinoma because circ-ITCH may exert an inhibitory effect on HCC^19^. Moreover, recent evidence also revealed the inhibitory effect of circ-ITCH in the progression of papillary thyroid cancer via affecting the circ-ITCH/miR-22-3p/CBL axis. In this study, we aimed to study the effect of rs4911154 on thyroid tumor malignancy and its underlying signaling pathways.

## Materials and methods

### Human subjects and sample collection

In this study, we recruited 202 patients of thyroid nodules and divided them into three groups according to the genotypes at rs4911154: 1. GG (N = 114); 2.GA (N = 72); 3. AA (N = 16). The basic characteristics including age, BMI, weight, height, fasting glucose, insulin, HOMA, GH, IGF1, IGFB3, total cholesterol, triglyceride, LDL, HDL, TSH and leptin were collected from the patients in all three groups and compared using one way ANOVA. The Human Research Ethics Committees of Lanzhou Maternal and Child Health Care Hospital has approved this research and all methods were performed in accordance with the last vision of the Declaration of Helsinki. Written informed consent was obtained from all patients before the study.

### Genotyping by direct sequencing

In this study, we collected 202 tissue samples of thyroid nodules, one from each patient diagnosed of thyroid nodules. To determine the genotypes at rs4911154 in each sample, genomic DNA of the samples was extracted and amplified by using PCR. Then, 2 μl of the PCR product obtained from each sample were added to 8 μl of a denaturing loading buffer, and PCR reaction was carried out for 10 min at 98 °C, followed by further reaction at 4 °C for 5 min. In the next step, 10 μl of sample from each patient were resolved by using a 10% polyacrylamide gel to ensure the integrity of the PCR product. Taqman genotyping assay (Applied Biosystems, Foster City, CA) was used to determine the GG, GA and AA genotypes of rs4911154 in each sample.

### RNA isolation and real-time PCR

In this study, real time PCR was used to measure the expression of circ-ITCH, miR-22-3p, and CBL mRNA in each sample. First, the total RNA content in each sample was separated by utilizing an RNeasy Mini assay kit (Qiagen, Valencia, CA) in accordance with the standard assay instructions provided in the assay protocol of the manufacturer’s assay kit manual. Then, the separated total RNA was actually reverse-transcribed into cDNA templates by using a RevertAid cDNA Synthesis assay kit (Thermo Fisher Scientific, Waltham, MA) in accordance with the standard assay instructions provided in the assay protocol of the manufacturer’s assay kit manual. In the next step, real time PCR reactions were conducted by using a SYBR Green master mix reagent (Invitrogen, Carlsbad, CA) in accordance with the standard assay instructions provided in the assay protocol of the manufacturer’s assay kit manual, and the real time PCR reactions were conducted on a 7500 real time PCR machine (Applied Biosystems, Foster City, CA) in accordance with the standard instructions provided in the instrument manufacturer. Finally, the 2^− ΔΔCT^ approach was adopted to compute the relative expression of circ-ITCH, miR-22-3p, and CBL mRNA in each sample.

### Cell culture and transfection

K1 and TPC-1 cells were purchased from ATCC and maintained in a DMEM medium (Gibco, Thermo Fisher Scientific, Waltham, MA) containing 10% of FBS (Clark Bio, Claymont, DE), 100 μg/ml of streptomycin, as well as 100 units/ml of penicillin. The culture conditions were 37 °C and 5% CO2. When K1 and TPC-1 cells reached confluency, they were used to establish several cellular models. In cell model I, K1 and TPC-1 cells were divided into four groups, i.e., (1) NC group (K1 and TPC-1 cells transfected with NC mimics); (2) miR-22-3p group (K1 and TPC-1 cells transfected with miR-22-3p mimics); (3) NC inhibitors group (K1 and TPC-1 cells transfected with NC inhibitors); and (4) miR-22-3p inhibitors group (K1 and TPC-1 cells transfected with miR-22-3p inhibitors). In cell model II, K1 and TPC-1 cells were also divided into four groups, i.e., (1) NC group (K1 and TPC-1 cells transfected with NC plasmids); (2) Circ-ITCH group (K1 and TPC-1 cells transfected with Circ-ITCH); (3) Circ-ITCH + NC siRNA group (K1 and TPC-1 cells transfected with both Circ-ITCH and NC siRNA); and (4) Circ-ITCH + CBL siRNA group (K1 and TPC-1 cells transfected with both Circ-ITCH and CBL siRNA). All transfections were carried out by using the Lipofectamine 3000 Transfection Reagent (Invitrogen, Carlsbad, CA) in accordance with the standard transfection instructions provided in the transfection protocol of the manufacturer’s manual, and the transfected cells were collected 48 h later for analysis.

### Cell proliferation assay

The proliferation of treated K1 and TPC-1 cells was measured using an MTT assay (Sigma Aldrich, St. Louis, MO) in accordance with the standard assay instructions provided in the assay protocol of the manufacturer’s assay kit manual, and the absorbance results were read at an emission wavelength of 490 nm using a microplate reader (Thermo Fisher Scientific, Waltham, MA) in accordance with the instructions provided in the instrument manual.

### Luciferase assay

Luciferase vectors containing wild type and mutant CBL were established: First, the wild type 3’ UTR of CBL containing the miR-22-3p binding site was inserted into a luciferase reporter vector pmiRGLO (Promega, Madison, WI) in accordance with the instructions for cloning provided in the manual of vector manufacturer. Then, a Quick Change mutagenesis assay kit was used to generate a site directed mutation in the miR-22-3p binding site of CBL 3’ UTR, and the mutant type 3’ UTR of CBL containing the mutated miR-22-3p binding site was also inserted into a luciferase reporter vector pmiRGLO. In the next step, K1 and TPC-1 cells were co-transfected with luciferase vectors of wild type or mutant CBL together with miR-22-3p. Similarly, our previous binding sites screening showed that miR-22-3p could potentially bind to circ-ITCH. Thus, luciferase vectors containing wild type and mutant circ-ITCH were established and transfected into K1 and TPC-1 cells with miR-22-3p. At 48 h post transfection, luciferase assays were carried out by using a Dual Luciferase reporter gene assay kit (Promega, Madison, WI) in accordance with the standard assay instructions provided in the assay protocol of the manufacturer’s assay kit manual to determine the luciferase expression of the cells.

### Western blot analysis

The protein expression of CBL in each tissue and cell sample was determined by using a Western blotting assay kit (Bio-Rad, Hercules, CA) in accordance with the standard assay instructions provided in the assay protocol of the manufacturer’s assay kit manual.

### Apoptosis analysis

In this study, the apoptosis of treated cells was assayed by using a BD FACS Canto II (BD Biosciences, Franklin Lakes, NJ) flow cytometer and an AnnexinV-FITC and PI assay kit (BD Biosciences, Franklin Lakes, NJ) in accordance with the standard assay instructions provided in the assay protocol of the manufacturer’s assay kit manual.

### Immunohistochemistry

The protein expression of CBL in each tissue sample was determined by using standard PFA-fixation, paraffin-embedding and ethanol dewaxing procedure, followed by incubation with anti-CBL antibody and a suitable secondary antibody subsequently in accordance with the incubation instructions provided in by the antibody manufacturer, and finally DAPI and hematoxylin staining. The tissue section images were analyzed by making use of a BX-51 microscopic (Olympus, Tokyo, Japan).

### TUNEL assay

In this study, we collected 202 tissue samples of thyroid nodules, one from each patient diagnosed of thyroid nodules. According to the genotypes at rs4911154, these tissue samples were divided into three groups: 1. GG (N = 114); 2.GA (N = 72); 3. AA (N = 16). Then, cells were isolated from tissue samples and the apoptosis status of the cells was determined by using a TUNEL assay kit (Roche, Indianapolis, IN) in accordance with the standard assay instructions provided in the assay protocol of the manufacturer’s assay kit manual.

### Statistical analysis

All results were shown as mean ± standard deviations (SD). Statistical analysis was done by utilizing SPSS 19.0 software (SPSS, Armonk, NY). Statistically significant differences were confirmed by a P value of < 0.05. All intergroup comparisons were done by utilizing one-way ANOVA.

## Results

### No obvious difference was observed in the basic characteristics of patients carrying different genotypes of rs4911154

In this study, we recruited 202 patients of thyroid nodules and grouped them according to the genotypes at rs4911154 as GG group (N = 114), GA group (N = 72) and AA group (N = 16). The basic characteristics including age, BMI, weight, height, fasting glucose, insulin, HOMA, GH, IGF1, IGFB3, total cholesterol, triglyceride, LDL, HDL, TSH and leptin were compared among the patients in the three groups and no obvious difference was observed (Table 1).

**Table 1:** Basic characteristics of different patient groups.

Characteristics	GG (N = 114)	GA (N = 72)	AA (N = 16)	*P* value
Age (years)	15.2 ± 3.7	14.4 ± 3.8	14.7 ± 3.2	0.347
BMI (kg/m^2^)	15.0 ± 2.5	15.1 ± 3.0	14.5 ± 2.8	0.747
Weight (kg)	13.5 ± 2.5	14.7 ± 1.8	13.9 ± 2.1	0.607
Height (cm)	12.5 ± 2.8	11.8 ± 2.1	12.3 ± 1.7	0.698
Fasting glucose (mg/dL)	14.8 ± 1.9	15.6 ± 2.8	15.1 ± 2.6	0.342
HOMA	14.5 ± 3.7	14.1 ± 3.6	13.6 ± 4.3	0.408
GH (ng/L)	11.0 ± 4.1	11.6 ± 4.5	10.7 ± 4.4	0.126
IGF1 (ng/L)	12.0 ± 4.2	11.8 ± 3.1	12.3 ± 4.9	0.659
IGFB3 (μg/L)	11.5 ± 3.2	11.1 ± 4.2	10.2 ± 4.2	0.588
Total cholesterol (mg/dL)	12.7 ± 2.4	12.4 ± 4.9	12.6 ± 3.7	0.405
Triglyceride (mg/dL)	13.1 ± 4.5	13.5 ± 4.3	12.9 ± 4.2	0.254
LDL (mg/dL)	13.8 ± 3.7	13.7 ± 4.8	13.1 ± 4.2	0.443
HDL (mg/dL)	12.2 ± 4.6	12.1 ± 4.1	12.6 ± 3.6	0.673
TSH (mg/dL)	12.2 ± 4.6	11.7 ± 3.4	11.4 ± 4.1	0.200
Leptin (ng/L)	12.8 ± 3.4	12.9 ± 4.4	12.3 ± 3.4	0.281

### The A allele of rs4911154 was correlated with a shortened doubling time of thyroid nodules

**Figure 1:**
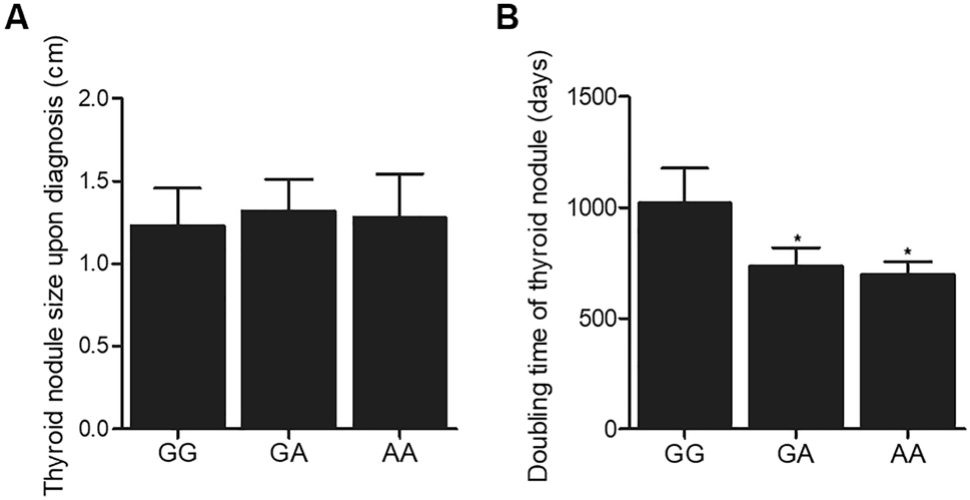
GG polymorphism at rs4911154 was correlated with increased doubling time of thyroid nodules (*P value < 0.05 vs. GG group). **(A)** No obvious difference was observed in the size of thyroid nodules in patients carrying different genotypes at rs4911154. **(B)** The doubling time of thyroid nodule was increased in patients carrying GG alleles when compared with patients carrying GA and AA alleles.

Ultrasound testing was performed to measure the size of thyroid nodules upon diagnosis and the doubling time of thyroid nodules. As shown in Fig. 1, there was no significant differences in repsect to the thyroid nodule size among the three patient groups (Fig. 1A). However, the doubling time of thyroid nodules in patients carrying the GA and AA genotype of rs4911154 was significantly shorter than patients carrying the GG genotype of rs491154 (Fig. 1B). Therefore, the presence of A allele might affect the doubling time rather than the size upon diagnosis of thyroid nodules.

### The GG genotype of rs4911154 was correlated with suppressed expression of miR-22-3p and elevated expression of circ-ITCH and CBL in the tissue samples of thyroid nodule patients

**Figure 2:**
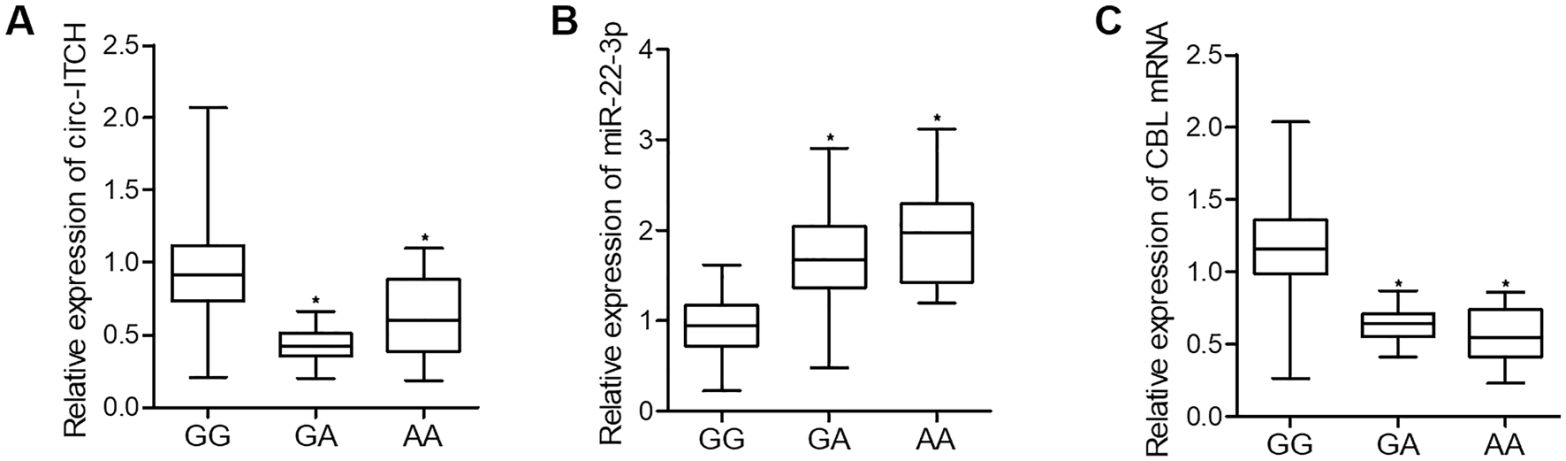
GG polymorphism at rs4911154 was correlated with increased expression of circ-ITCH and CBL mRNA but decreased expression of miR-22-3p in the tissue samples in thyroid nodule patients (*P value < 0.05 vs. GG group). **(A)** The expression of circ-ITCH was increased in patients carrying GG alleles at rs4911154 when compared with patients carrying the GA and AA genotypes. **(B)** The expression of miR-22-3p was decreased in patients carrying GG alleles at rs4911154 when compared with patients carrying the GA and AA genotypes. **(C)** The expression of CBL mRNA was increased in patients carrying GG alleles at rs4911154 when compared with patients carrying the GA and AA genotypes.

**Figure 3:**
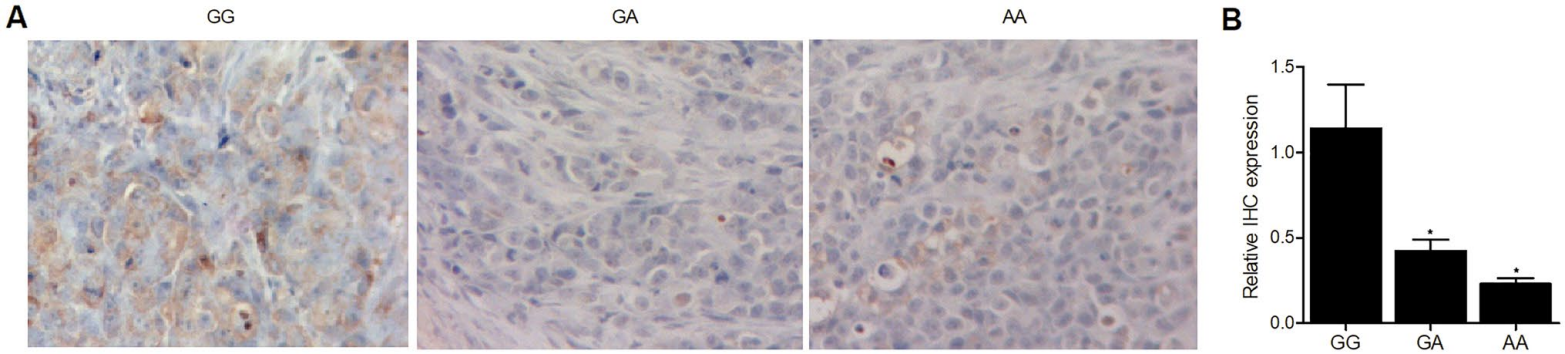
Immunohistochemistry analysis showed that GG polymorphism at rs4911154 was correlated with increased expression of CBL protein in the tissue samples of thyroid nodule patients (*P value < 0.05 vs. GG group).

To further explore the effect of rs4911154 polymorphism, we further investigated the relative expression level of circ-ITCH, miR-22-3p and CBL mRNA in thyroid nodule tissue samples collected from different patient groups. As shown in Fig. 2, the relative expression levels of circ-ITCH (Fig. 2A) and CBL mRNA (Fig. 2C) were both remarkably decreased in the thyroid nodule tissue samples of patients carrying the A allele at rs4911154 when compared with patients with GG genotype of rs4911154. On the contrary, the relative expression level of miR-22-3p was notably promoted with the presence of A allele compared with the mere presence of G allele at rs4911154 (Fig. 2B). Moreover, as shown in Fig. 3, IHC analysis upon CBL protein expression also showed decreased CBL protein level with the presence of allale A at rs4911154. Therefore, it can be suggested that the presence of allele A at rs4911154 is associated with down-regulated circ-ITCH and CLB and up-regulated miR-22-3p.

### The GG genotype at rs4911154 was correlated with an elevated rate of tissue apoptosis

**Figure 4:**
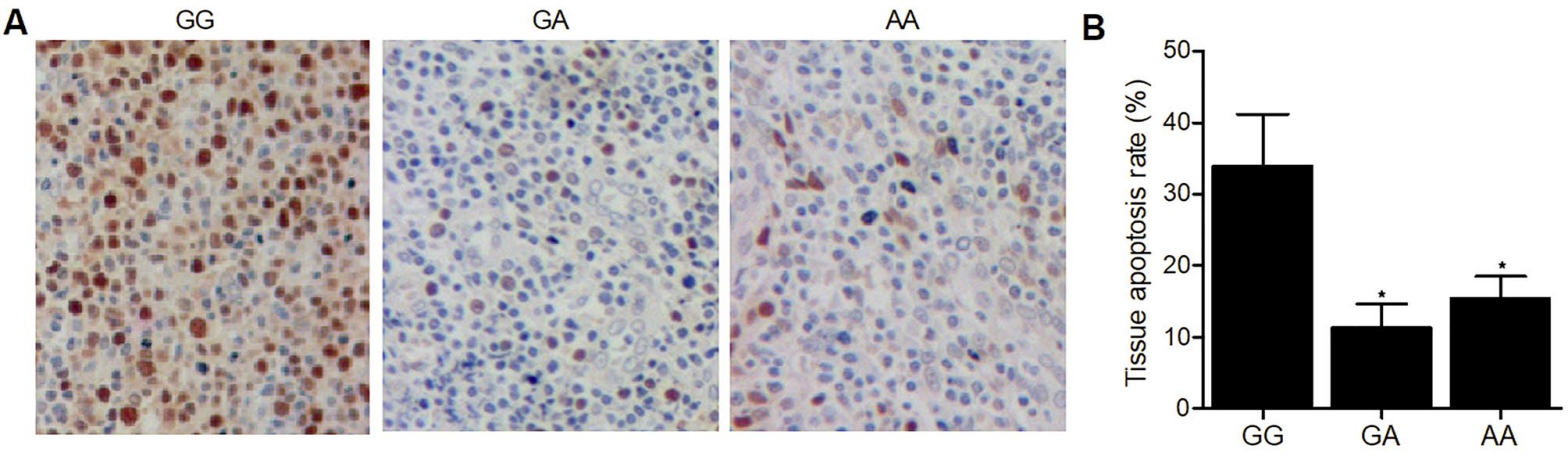
TUNEL analysis showed that GG polymorphism at rs4911154 was correlated with an increased apoptosis rate in the tissue samples of thyroid nodule patients (*P value < 0.05 vs. GG group).

Moreover, TUNEL was carried out to assess the effect of rs4911154 polymorphism on the cellular apoptosis in tissue samples of thyroid nodule patients (Fig. 4A). Accordingly, the tissue apoptosis rate (Fig. 4B) was apparently suppressed in thyroid patients carrying the GA and AA genotypes when compared with patients carrying the GG genotype (Fig. 4), indicating allele A is associated with suppressed tissue apoptosis.

### CBL could bind to miR-22-3p through its binding site on 3’ UTR

**Figure 5:**
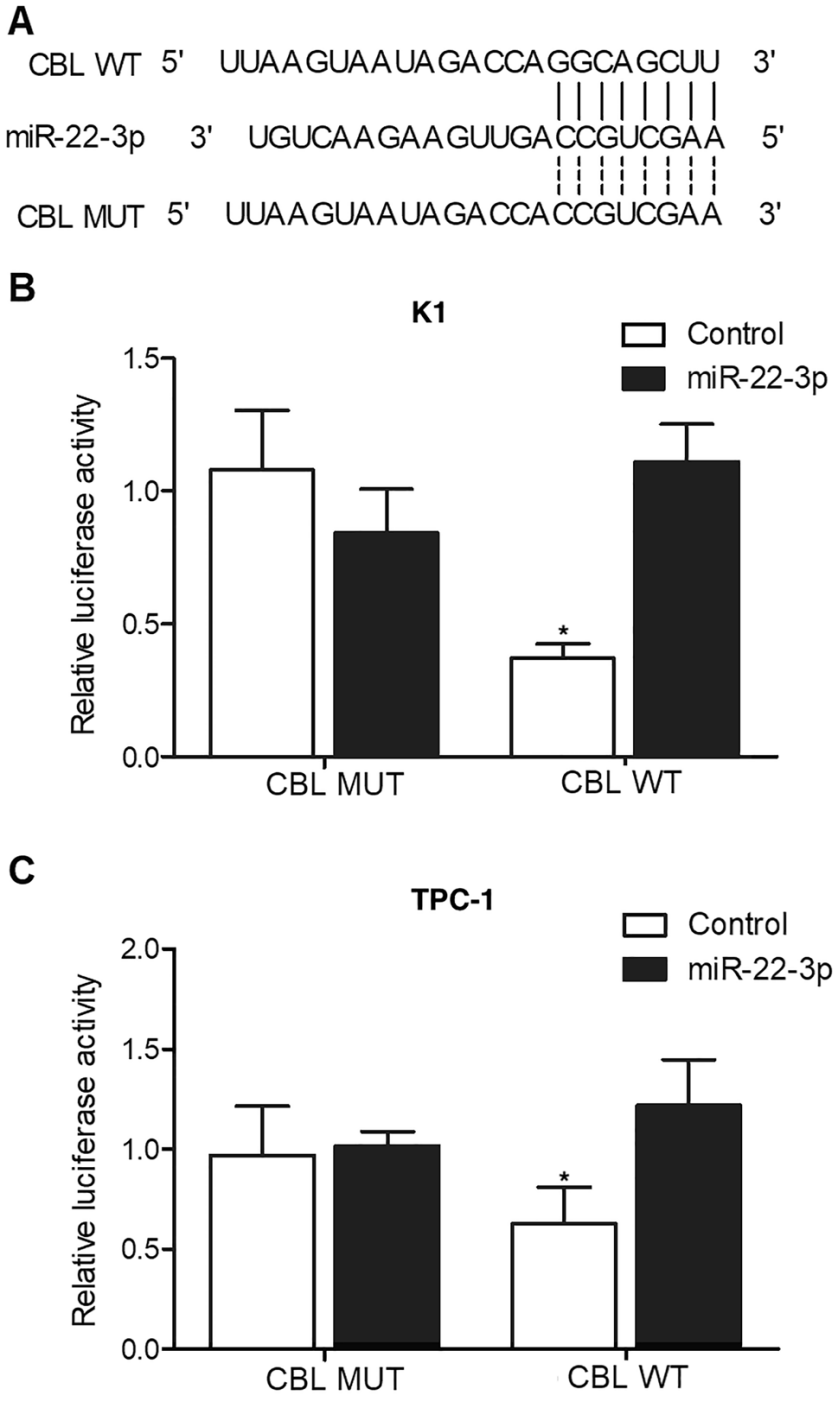
MiR-22-3p suppressed the expression of CBL through binding to its 3’ UTR (*P value < 0.05 vs. control). **(A)** Sequence analysis indicated binding of miR-22-3p to the 3’ UTR of CBL. **(B)** The luciferase activity of wild type CBL was inhibited by miR-22-3p in K1 cells. **(C)** The luciferase activity of wild type CBL was inhibited by miR-22-3p in TPC-1 cells.

Binding sites screening showed that miR-22-3p could potentially bind to the 3’ UTR of CBL (Fig. 5A). Therefore, luciferase vectors containing wild type and mutant CBL 3’UTR were constructed and transfected into K1 (Fig. 5B) and TPC-1 cells (Fig. 5C) along with miR-22-3p to study their regulatory relationships. Accordingly, the luciferase activity was evidently suppressed in K1 cells co-transfected with wild type CBL 3’UTR and miR-22-3p compared with other cell groups (Fig. 5B), and similar results were obtained when repeating the observation in TPC-1 cells (Fig. 5C). Therefore, it can be conclude that CBL is a target gene of miR-22-3p.

### Alteration of miR-22-3p in K1 and TPC-1 cells regulated the expression of CBL mRNA and protein

**Figure 6:**
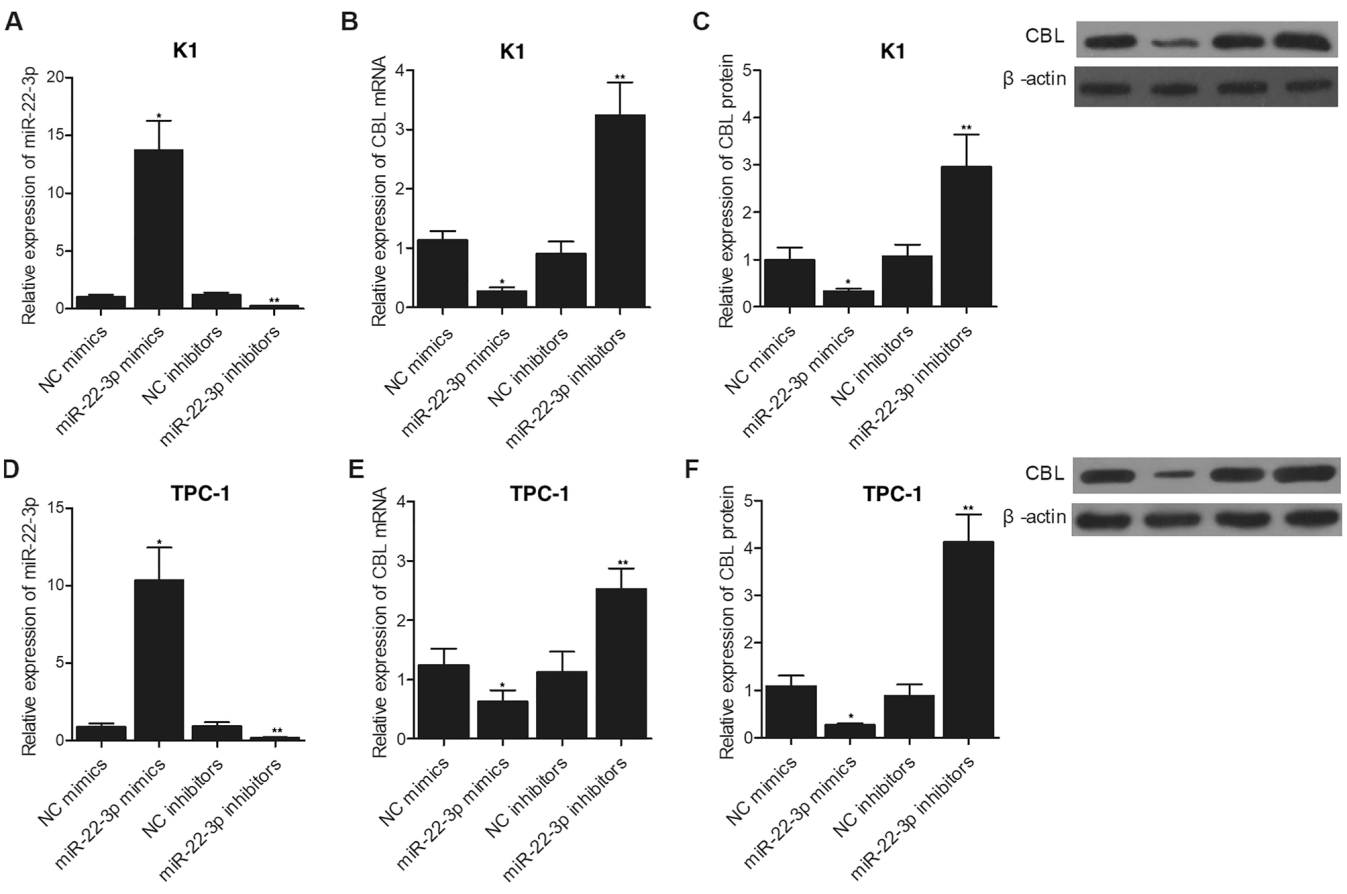
CBL expression was negatively correlated with miR-22-3p expression in K1 and TPC-1 cells (*P value < 0.05 vs. NC mimics; **P value < 0.05 vs. NC inhibitors). **(A)** The expression of miR-22-3p was enhanced by miR-22-3p mimics and suppressed by miR-22-3p inhibitors in K1 cells. **(B)** The expression of CBL mRNA was suppressed by miR-22-3p mimics and activated by miR-22-3p inhibitors in K1 cells. **(C)** The expression of CBL protein was suppressed by miR-22-3p mimics and activated by miR-22-3p inhibitors in K1 cells. **(D)** The expression of miR-22-3p was enhanced by miR-22-3p mimics and suppressed by miR-22-3p inhibitors in TPC-1 cells. **(E)** The expression of CBL mRNA was suppressed by miR-22-3p mimics and activated by miR-22-3p inhibitors in TPC-1 cells. **(F)** The expression of CBL protein was suppressed by miR-22-3p mimics and activated by miR-22-3p inhibitors in TPC-1 cells.

MiR-22-3p mimics and inhibitors were respectively transfected into K1 and TPC-1 cells to study the regulatory relationship between miR-22-3p and CBL. As shown in Fig. 6, the overexpression of miR-22-3p induced by miR-22-3p mimics (Fig. 6A) in K1 cells significantly suppressed the expression of CBL mRNA (Fig. 6B) and protein (Fig. 6C), while the knockdown of miR-22-3p induced by miR-22-3p inhibitors (Fig. 6A) in K1 cells evidently promoted the expression of CBL mRNA (Fig. 6B) and protein (Fig. 6C). Similarly, when repeating the above observation in TPC-1 cell (Fig. 6D–F), same results in respect to the expression of CBL mRNA and protein were obtained, suggesting the establishment of the negative regulatory relationship between miR-22-3p and CBL.

### Circ-ITCH was targeted by miR-22-3p

**Figure 7:**
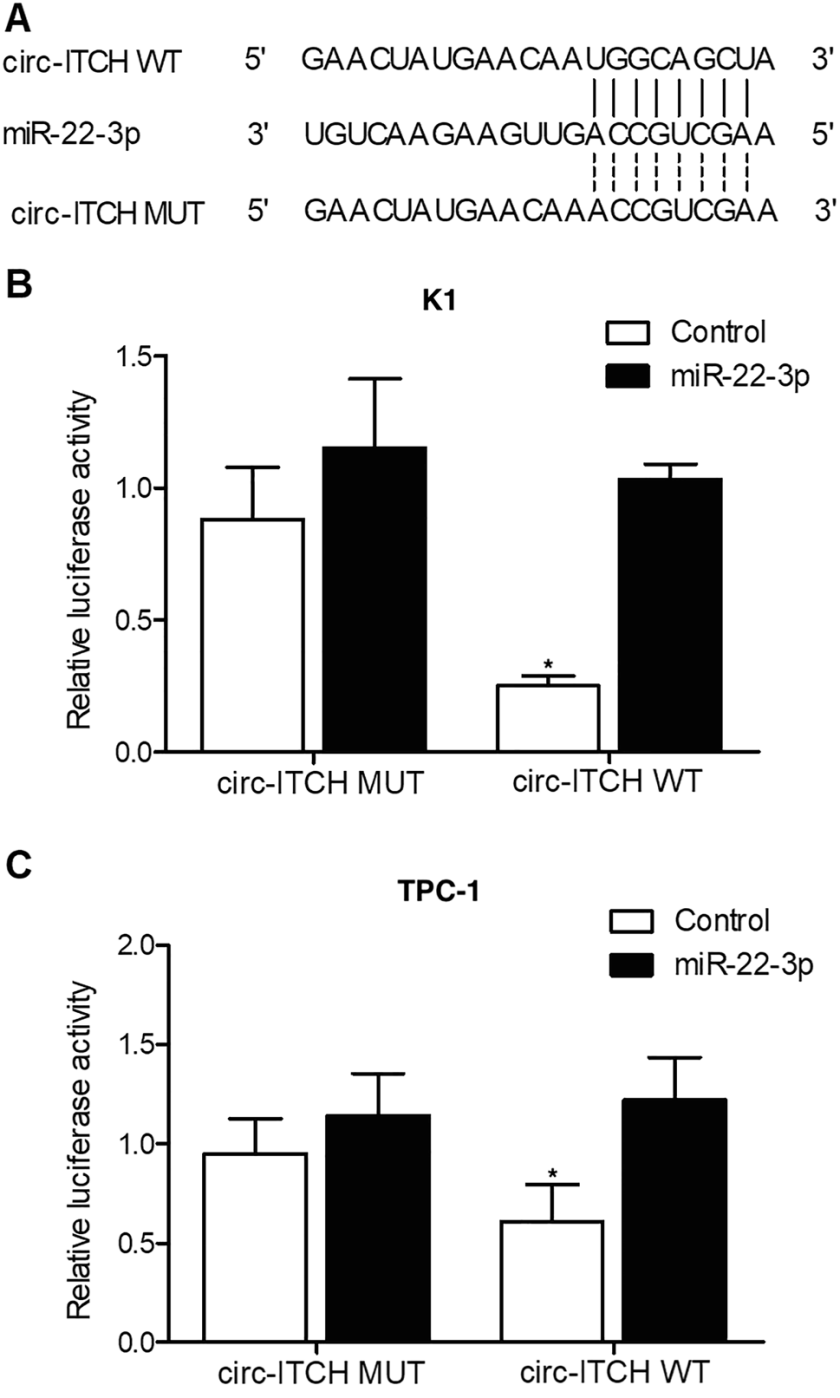
MiR-22-3p suppressed the expression of circ-ITCH (*P value < 0.05 vs. control). **(A)** Sequence analysis indicated binding of miR-22-3p to circ-ITCH. **(B)** The luciferase activity of wild type circ-ITCH was inhibited by miR-22-3p in K1 cells. **(C)** The luciferase activity of wild type circ-ITCH was inhibited by miR-22-3p in TPC-1 cells.

Sequence analysis indicated that miR-22-3p could potentially target circ-ITCH (Fig. 7A) on specific binding sites. Therefore, luciferase vectors containing wild type and mutant circ-ITCH were constructed and transfected into K1 (Fig. 7B) and TPC-1 (Fig. 7C) cells with miR-22-3p. Accordingly, the luciferase activity was effectively suppressed in K1 (Fig. 7B) or TPC-1 (Fig. 7C) cells co-transfected with wild type circ-ITCH and miR-22-3p, thus verifying that circ-ITCH could bind to miR-22-3p.

### CBL siRNA abolished circ-ITCH induced CBL up-regulation in K1 and TPC-1 cells

**Figure 8:**
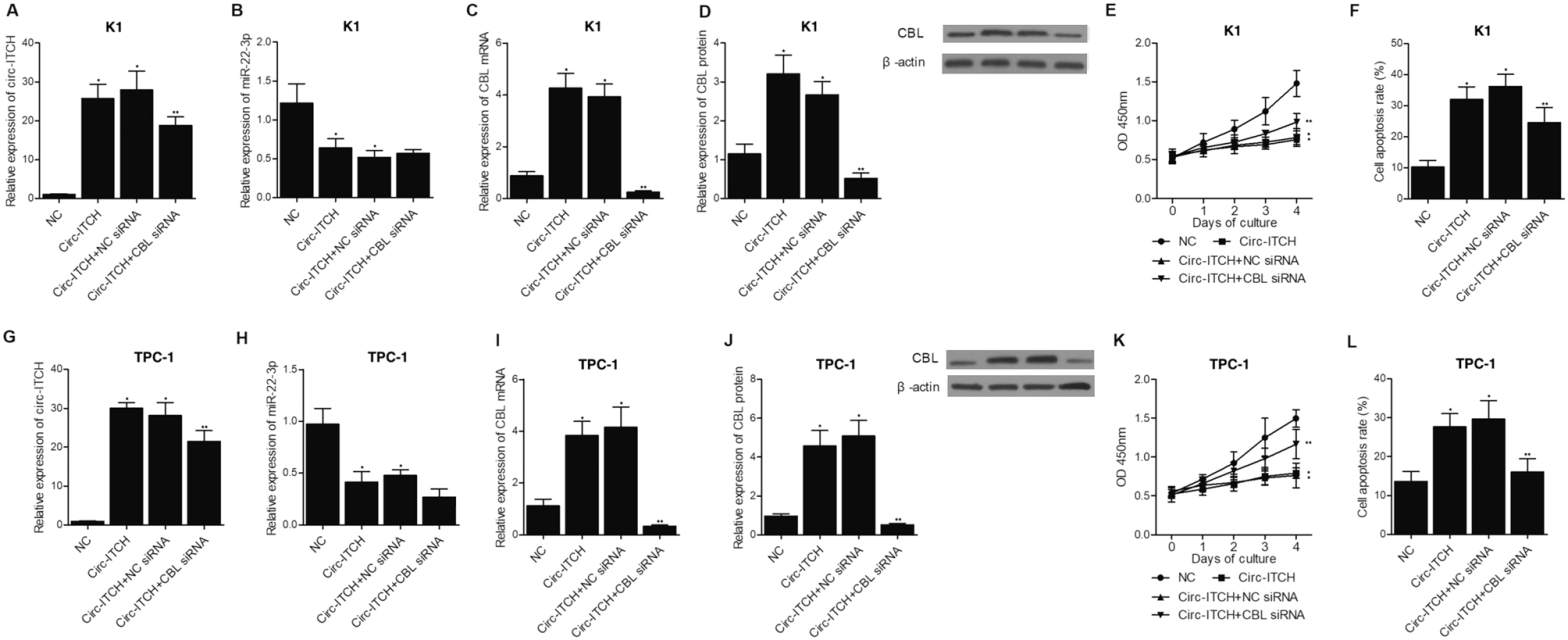
CBL siRNA abolished circ-ITCH-induced up-regulation of CBL, reduction of cell proliferation and elevation of apoptosis rate (*P value < 0.05 vs. NC; **P value < 0.05 vs. circ-ITCH + NC siRNA). **(A)** CBL siRNA decreased the up-regulation of circ-ITCH expression in K1 cells transfected with circ-ITCH plasmids. **(B)** The expression of miR-22-3p was remarkably suppressed in K1 cells transfected with circ-ITCH plasmids. **(C)** The circ-ITCH induced up-regulation of CBL mRNA was inhibited by CBL siRNA in K1 cells. **(D)** The circ-ITCH induced up-regulation of CBL protein was inhibited by CBL siRNA in K1 cells. **(E)** The circ-ITCH induced decrease of cell proliferation was maintained by CBL siRNA in K1 cells. **(F)** The circ-ITCH induced elevation of apoptosis rate was restored by CBL siRNA in K1 cells. **(G)** CBL siRNA decreased the up-regulation of circ-ITCH expression in TPC-1 cells transfected with circ-ITCH plasmids. **(H)** The expression of miR-22-3p was remarkably suppressed in TPC-1 cells transfected with circ-ITCH plasmids. **(I)** The circ-ITCH induced up-regulation of CBL mRNA was inhibited by CBL siRNA in TPC-1 cells. **(J)** The circ-ITCH induced up-regulation of CBL protein was inhibited by CBL siRNA in TPC-1 cells. **(K)** The circ-ITCH induced decrease of cell proliferation was maintained by CBL siRNA in TPC-1 cells. **(L)** The circ-ITCH induced elevation of apoptosis rate was restored by CBL siRNA in TPC-1 cells.

To study the regulatory relationship between circ-ITCH and miR-22-3p and accordingly establish the signaling pathway which connects circ-ITCH, miR-22-3p and CBL, circ-ITCH plasmids were transfected into K1 cells in combination with CBL siRNA. As shown in Fig. 8, remarkable elevation of circ-ITCH was observed in K1 cells transfected with circ-ITCH plasmids, while the co-transfection of CBL siRNA showed limited effect on reversing the up-regulation of circ-ITCH in K1 cells (Fig. 8A). Meanwhile, relative expression of miR-22-3p was generally inhibited in K1 cells transfected with circ-ITCH, circ-ITCH + NC siRNA and circ-ITCH + CBL siRNA (Fig. 8B). Also, the up-regulation of circ-ITCH significantly activated the expression of CBL mRNA (Fig. 8C) and protein (Fig. 8D) in K1 cells. However, CBL siRNA abolished circ-ITCH-induced CBL up-regulation to a level even lower than in the control cells. And subsequent MTT assay and flow analysis also showed that CBL siRNA could effectively restore circ-ITCH-induced decrease of K1 cell proliferation (Fig. 8E) and reverse circ-ITCH stimulated elevation of K1 cell apoptosis (Fig. 8F). All the results observed in K1 cells were independently confirmed in TPC-1 cells (Fig. 8G–L). Therefore, it can be concluded that a signaling pathway of circ-ITCH/miR-22-3p/CBL is established, and is as well associated with cell proliferation and apoptosis.

## Discussion

In this study, we recruited 202 thyroid nodule patients and divided them into three groups according to their genotypes at rs4911154. Ultrasound was performed to analyze the thyroid nodule size and the doubling time of thyroid nodules. The GG genotype significantly increased the doubling time of thyroid nodules. In addition, we compared the expression of circ-ITCH, miR-22-3p and CBL in the tissue samples from thyroid nodule patients carrying different alleles at rs4911154. The expression of circ-ITCH and CBL was remarkably elevated, while the expression of miR-22-3p was notably suppressed in the tissue samples of thyroid nodule patients carrying the GG genotype when compared with patients carrying the GA and AA alleles.

Past research showed the function of circ-ITCH in the progression as well as development of HCC, and showed that rs10485505 as well as rs4911154 SNPs were closely linked to enhanced risk of HCC. It was additionally found that the rs4911154 and rs10485505 SNPs in the circITCH gene were considerably linked to higher HCC risks. This research suggests that circ-ITCH can be used as a prognostic biomarker for HCC^19^.

Circ-ITCH downregulation was reported in esophageal squamous cell cancer, colorectal cancer, lung carcinoma as well as hepatocellular carcinoma by sponging ITCH targeting miRNAs^20–22^. Circ-ITCH also acts as a tumor suppressor in breast cancer through sponging miR-224 and miR-17, or in lung cancer through sponging miR-224 and miR-7 and in esophageal cancer through sponging miR-17 miR-7, and miR-214^21–23^. In this study, we performed luciferase assay to explore the regulatory effect of miR-22-3p on CBL as well as on circ-ITCH in K1 and TPC-1 cells. The luciferase activities of wild type CBL and circ-ITCH were significantly suppressed by miR-22-3p in K1 and TPC-1 cells. The unique feature of circ-ITCH was revealed in PTC progression, i.e., the circ-ITCH/miR-22-3 p/CBL/b-catenin pathway can restrain PTC development^13^.

Bioinformatics analyses as well as luciferase assays illustrated that circ-ITCH can sponge miR-22-3p to upregulate CBL expression. Raised expression of CBL subdued the activation of Wnt/b-catenin signaling to result in hindered PTC development^13^. It was also shown that miRNA-22-3p changed markedly in cervical carcinoma to regulate its growth. Based upon these data, it was hypothesized that miR-22-3p is closely linked to the onset of cervical cancer. Previous researches have presented that miR-22-3p is often dysregulated in human illness. For instance, miR-22-3p is upregulated in prostatic cancer, colorectal cancer, as well as endometriosis but downregulated in esophageal cancer^24–26^. On top of that, miR-22-3p can target various genes to either suppress or promote tumour progression. For instance, miR-513a-5p, miR-22-3p as well as miR-625-5p expression is downregulated in asthma patients due to their close association to cytokine release and immune response. Also regulated by miR-22-3p and miR-513a-5p, CBL is significantly linked to B cell and T cell differentiation^27^. In this study, we altered the expression of miR-22-3p in K1 and TPC-1 cells, and found that the expression of CBL mRNA and protein was negatively correlated with the expression of miR-22-3p in K1 and TPC-1 cells.

CBL-b is composed of a RING finger domain, a Src homology 2 domain, as well as a proline-rich domain carrying sites for tyrosine phosphorylation. Acting as an E3 ubiquitin ligase, CBL’s RING finger domain can recruit E2, an enzyme that can conjugate to ubiquitin, while the Src homology 2 domain of CBL can identify proteins for ubiquitin conjugation^28–30^. The neddylation process mediated by C-CBL appears to work against the activity of c-Src in promoting the metastasis of glioblastoma as well as lung cancer. Thus, neddylation might be used as a target to protect against cancer metastasis^31^. Working as a ligase of ubiquitin, CBL-b is associated with T cell and B cell regulation as well as the activation of mast cells^32–34^. It was shown that CBL-b is needed for the apoptosis activation of T cells. It was also uncovered that CBL-b can reverse multi-drug resistance through inhibiting the activation of Akt in stomach cancer^35^. In this study, we transfected CBL siRNA into K1 and TPC-1 cells, in which the expression of circ-ITCH was activated. CBL siRNA effectively abolished circ-ITCH-overexpression-induced CBL up-regulation and cellular proliferation reduction, as well as the elevation in the apoptosis rate of K1 and TPC-1 cells.

However, the results obtained were limited. Apart from human study, establishing an animal model to test the involvement of circ-ITCH/miR-22-3p/CBL axis in the development of thyroid cancer is also preferred. Therefore, in our future study, an animal model will be utilized to further verify our findings.

## Conclusion

As indicated by the results, a signaling pathway of circ-ITCH/miR-22-3p/CBL axis was established to explain the effect of SNP of circ-ITCH on thyroid tumor malignancy. Compared with the G allele, the A allele in rs4911154 contributed to the malignancy of thyroid nodules by decreasing doubling time and down-regulating CBL expression (Suppl. Information).

## Supplementary Information

Supplementary Figures.

## Data Availability

The data that support the findings of this study are available from the corresponding author upon reasonable request.
