# Supplementary material for: Rs4911154 of circ-ITCH aggravated tumor malignancy of thyroid nodules via the circ-ITCH/miR-22-3p/CBL axis
Source: Sci Rep. 2021 Sep 16;11:18491. doi: 10.1038/s41598-021-97471-5 (PMC8445954; doi:10.1038/s41598-021-97471-5)

Supplementary Files for Rs4911154 of circ-ITCH aggravated tumor malignancy of thyroid nodules via the circ-ITCH/miR-22-3p/CBL axis

Original Image for Fig 6C CBL

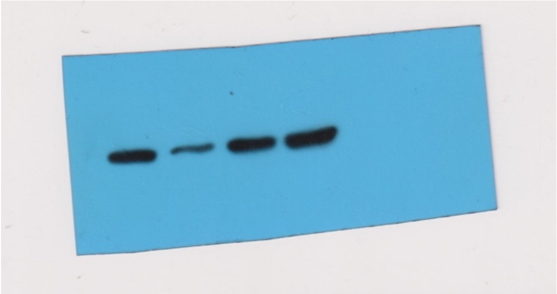

Original Image for Fig 6C beta actin

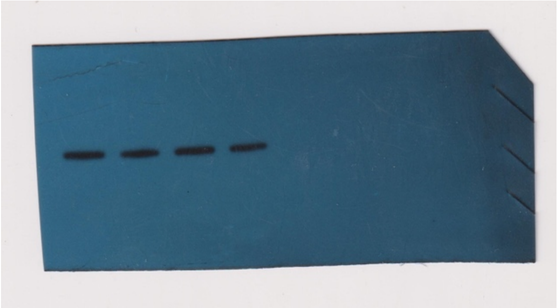

Original Image for Fig 6F CBL

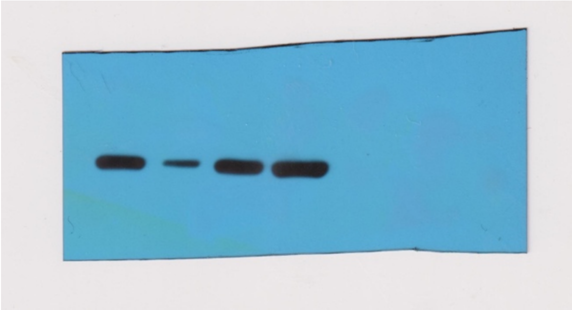

Original Image for Fig 6F beta actin

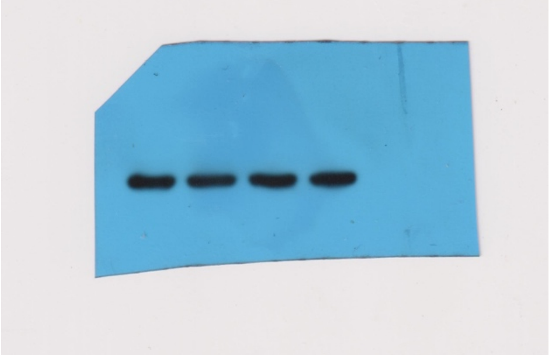

Original Image for Fig 8D CBL

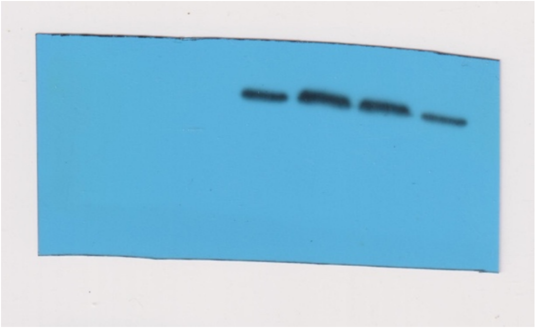

Original Image for Fig 8D beta actin

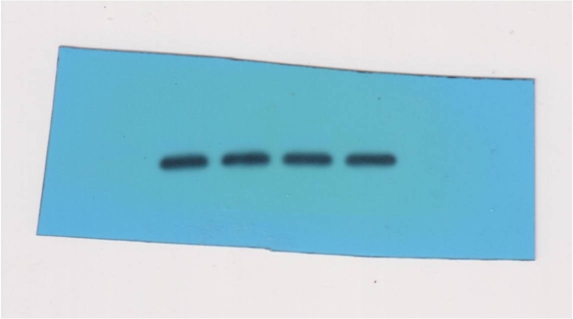

Original Image for Fig 8J CBL

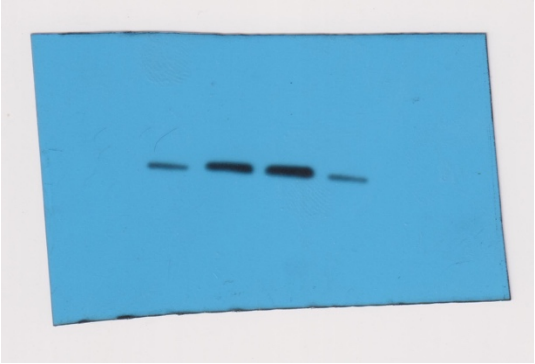

Original Image for Fig 8J beta actin

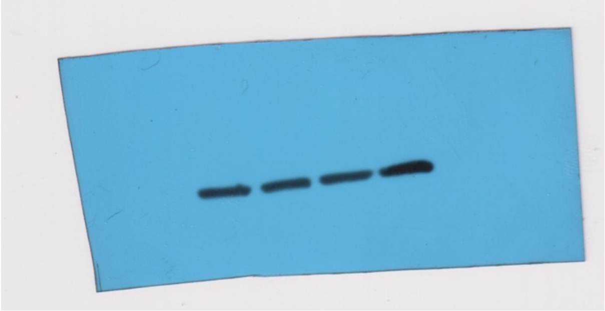

Supplement: Supplementary file 1 — Supplementary Figures. [file 41598_2021_97471_MOESM1_ESM.pdf]
